# Cause of death and influencing factors of chronic renal failure on maintenance hemodialysis

**DOI:** 10.12669/pjms.39.5.7037

**Published:** 2023

**Authors:** Xing Fan, Jing Li, Zhao-yu Bi, Wei Liang, Feng-juan Wang

**Affiliations:** 1 Xing Fan Department of Nephrology, Affiliated Hospital of Hebei University, Baoding 071000, Hebei China. Key Laboratory of Bone Metabolism and Physiology in Chronic Kidney Disease of Hebei Province, Baoding 071000, Hebei China; 2 Jing Li Department of Nephrology, Affiliated Hospital of Hebei University, Baoding 071000, Hebei China. Key Laboratory of Bone Metabolism and Physiology in Chronic Kidney Disease of Hebei Province, Baoding 071000, Hebei China; 3 Zhao-yu Bi Department of Internal Medicine, Affiliated Hospital of Hebei University, Baoding 071000, Hebei China. Key Laboratory of Bone Metabolism and Physiology in Chronic Kidney Disease of Hebei Province, Baoding 071000, Hebei China; 4 Wei Liang Department of Nephrology, Affiliated Hospital of Hebei University, Baoding 071000, Hebei China. Key Laboratory of Bone Metabolism and Physiology in Chronic Kidney Disease of Hebei Province, Baoding 071000, Hebei China; 5 Feng-juan Wang Supply Room, Affiliated Hospital of Hebei University, Baoding 071000, Hebei China. Key Laboratory of Bone Metabolism and Physiology in Chronic Kidney Disease of Hebei Province, Baoding 071000, Hebei China

**Keywords:** Chronic Renal Failure, Maintenance Hemodialysis, Death, Influencing Factors

## Abstract

**Objective:**

To investigate the causes of death in patients with chronic renal failure (CRF) on maintenance hemodialysis and its influencing factors.

**Methods:**

This is a retrospective study. A total of 300 patients with chronic renal failure undergoing maintenance hemodialysis who were admitted to the Affiliated Hospital of Hebei University from March 2020 to October 2022 were selected as subjects. Various information of patients were collected. In addition, 80 dead patients in this group were investigated for the cause of death, including cardiovascular and cerebrovascular diseases, infections, multi organ failure, and other causes, and the death-related conditions of cardiovascular and cerebrovascular diseases, such as triglyceridr,,total cholesterol, and in blood lipid levels were analyzed.

**Results:**

Among the 80 dead patients, cardiovascular and cerebrovascular diseases accounted for a higher proportion of death (66%). Univariate Logistic regression analysis showed that advanced age, plasma homocysteine, blood parathyroid hormone, hyperphosphatemia, hypertension, high volume load and left ventricular hypertrophy were risk factors for death in patients with chronic renal failure on maintenance hemodialysis. Multivariate Logistic regression analysis showed that high volume load, left ventricular hypertrophy and anemia were risk factors for death on maintenance hemodialysis. The levels of hemoglobin (HGB) and high-density lipoprotein (HDL) in patients who died of cardiovascular and cerebrovascular diseases were significantly lower than those in the non-cardio-cerebrovascular death group (P=0.00), and the levels of serum phosphorus, TG and TC were significantly higher than those in the non-cardio-cerebrovascular death group (P, P=0.00; TG, P=0.02; TC, P=0.01).

**Conclusion:**

Cardiovascular and cerebrovascular diseases are the leading cause of death in patients with chronic renal failure on maintenance hemodialysis. Adequate dialysis and normal hemoglobin levels are favorable protective factors.

## INTRODUCTION

Chronic renal failure (CRF) is a loss of renal function caused by various kidney diseases and an irreversible pathological change.^1^ Clinically, alternative therapies, including kidney transplantation, peritoneal dialysis and hemodialysis, are the preferred treatment modalities for CRF.^2^ Kidney transplantation is the gold standard for the treatment of end-stage renal failure with clear benefits.^3^ However, there are certain limitations, such as donor shortage, high treatment cost, obvious postoperative complications and rejection reaction, and the inability of long-term treatment for patients due to tolerance to immunosuppressive agents or severe adverse reactions.^4^ In recent years, rapid development has been made in hemodialysis technology, and patients undergoing hemodialysis have had an ever-improving survival rate and quality of life.^5^ Nevertheless, there are still many clinical complications in patients, which seriously affect the dialysis effect and quality of life, coupled with a higher mortality rate from dialysis-related complications, especially cardiovascular and cerebrovascular diseases and infections.^6^ In this study, the causes of death in patients with CRF on maintenance hemodialysis were investigated and its influencing factors were analyzed, in order to provide a reference for targeted preventive measures in clinic.

## METHODS

This is a retrospective study. A total of 300 patients with chronic renal failure undergoing maintenance hemodialysis who were admitted to the Affiliated Hospital of Hebei University from March 2020 to October 2022 were selected as subjects.

### Ethical Approval

This study has been approved by the Medical Ethics Committee of Ethical Approval: Affiliated Hospital of Hebei University on December 21, 2022(No.:HDFYLL-KY-2022-019), and written informed consent was obtained from all participants.

### Inclusion criteria:

- Patients who dialysis time >three months;
- Patients who had underwent hemodialysis and died;
- Patients whose family members were willing to cooperate in completing the questionnaire survey;
- Patients with complete clinical data.

### Exclusion criteria:

- Patients with acute kidney injury and chronic kidney disease patients not requiring hemodialysis;.
- Patients with dialysis time < three months;
- Patients whose family members were unwilling to cooperate with the study.

All patients were followed up, and their various information were collected, including gender, age, occupation, marital status, education level, height, body weight, hemoglobin, serum creatinine, blood urea nitrogen, serum calcium, serum phosphorus, serum albumin, serum cholesterol, triacylglycerol, high-density lipoprotein, low-density lipoprotein, very low-density lipoprotein, blood parathyroid hormone level, plasma homocysteine level, blood pressure and heart rate after last hemodialysis, volume status, left ventricular muscle hypertrophy, adequate dialysis or not, underlying diseases, etc.

Of the 300 patients enrolled in this study, 80 died and were analyzed as follows:

The cause of death were investigated, including cardiovascular and cerebrovascular diseases (cerebral hemorrhage, cerebral infarction, heart failure, myocardial infarction, arrhythmia, sudden cardiac death), infection (pulmonary infection, urinary tract infection, catheter-related infection, other infections), multiple organ failure, and other causes

 The death-related conditions of cardiovascular and cerebrovascular diseases, such as TG, TC, and in blood lipid levels were analyzed.

### Statistical analysis

All data in this study were counted by SPSS 20.0 software. The measurement data were expressed as (*χ̅*±*S*), and the comparison of counting data was expressed as absolute value or composition ratio. Two independent samples t-test was used for data analysis between groups, χ^2^ test was used for rate comparison, and Logistic regression analysis was used for influencing factors. P<0.05 indicates a statistically significant difference.

## RESULTS

Among the 80 dead patients, the leading cause of death was cardiovascular disease, followed by cerebrovascular disease and infection (Table-I). The results of univariate Logistic regression analysis suggest that advanced age, plasma homocysteine, blood parathyroid hormone, hyperphosphatemia, hypertension, high volume load and left ventricular hypertrophy were risk factors for death in patients with chronic renal failure on maintenance hemodialysis. In contrast, good dialysis and normal HBG content (HBG≥90g/L) were favorable protective factors for death in patients with chronic renal failure on maintenance hemodialysis (Table-II).

**Table-I:** Cause of death in patients with chronic renal failure on maintenance hemodialysis (*χ̅*±*S*) n=80.

Cause of death	Number of cases	Composition ratio (%)	Sorting order
Cardiovascular diseases	32	40%	1
Heart failure	12	15%	
Myocardial infarction	9	11%	
Arrhythmia	7	9%	
Sudden cardiac death	4	5%	
Cerebrovascular disease	21	26%	2
Intracerebral hemorrhage	13	16%	
Cerebral infarction	8	10%	
Infection	18	23%	3
Lung infection,	9	11%	
Urinary tract infection,	6	8%	
Catheter-related infections	3	4%	
Multiple organ failure	5	6%	4
Tumor	4	5%	5

**Table-II:** Univariate Logistic regression analysis of death in patients with chronic renal failure on maintenance hemodialysis.

Influencing factors	Assignment	Β value	SE value	Waldχ^2^	P value	OR value	95.0% CI
Age	≤70 years old=0 >70 years old=1	1.777	0.806	4.866	0.027	5.912	1.219~28.671
Blood homocysteine	<16 umol/L=0, ≥16 umol/L=1	2.438	0.896	7.396	0.007	11.450	1.976~66.354
Parathyroid hormone	<300ng/L=0, ≥300ng/L=1	1.887	0.847	4.971	0.026	6.602	1.256~34.696
Hyperphosphatemia	No=0, Yes=1	2.021	0.837	5.830	0.016	7.549	1.463~38.950
Hypertension	No=0, Yes=1	2.169	0877	6.114	0.013	8.747	1.568~48.806
High volume load	No=0, Yes=1	2.083	1.001	4.327	0.038	8.026	1.128~57.113
Left ventricular hypertrophy	No=0, Yes=1	2.745	0.851	10.404	0.001	15.559	2.936~82.464
Hemoglobin	<90g/L=0, ≥90g/L=1	—2.881	0.962	8.975	0.003	0.056	0.009~0.369
Dialysis situation	Insufficient=0, Sufficient=1	—3.244	0.839	14.946	0.000	0.039	0.008~0.202

p<0.05.

The patients’ death was taken as the dependent variable, and the eight independent variables entered into the univariate Logistic regression equation of chronic renal failure maintenance hemodialysis death were taken as dependent variables. Multivariate logistic regression analysis was performed after assignment, α _in_ = 0.05, α _out_ = 0.10. The results suggest that high volume load, left ventricular hypertrophy and anemia were risk factors for death on maintenance hemodialysis. In contrast, adequate hemodialysis is a protective factor for death in patients with chronic renal failure on maintenance hemodialysis (Table-III).

**Table-III:** Univariate Logistic regression analysis of death in patients with chronic renal failure on maintenance hemodialysis.

Influencing factors	Assignment	Β value	SE value	Waldχ^2^	P value	OR value	95.0% CI
High volume load	No=0, Yes=1	-2.627	0.506	26.947	0.000	0.0724	0.027~20.195
Left ventricular Hypertrophy	No=0, Yes=1	-2.565	0.506	25.655	0.000	0.077	0.029~0.208
Anemia	No=0, Yes=1	-1.365	0.435	9.834	0.002	0.255	0.109~0.599
Dialysis situation	Insufficient=0, Sufficient=1	2.971	0.492	36.495	0.000	19.518	7.444~51.181

p<0.05.

The analysis of death from cardio-cerebrovascular diseases in patients with chronic renal failure on maintenance hemodialysis showed that the patients who died with cardio-cerebrovascular diseases were found to have short duration of time since they were started on hemodialysis than that of patients without cardio-cerebrovascular diseases, the level of HBG was significantly lower than that in the non-cardio-cerebrovascular death group, and P, TG and TC were significantly higher than those in the non-cardiovascular death group, with statistically significant differences (P<0.05). While the level of HDL was significantly lower than that in the non-cardiovascular death group (P=0.00) (Table-IV).

**Table-IV:** Analysis of death from cardiovascular and cerebrovascular diseases in patients with chronic renal failure on maintenance hemodialysis (*χ̅*±*S*).

Observation indicator	Cardiovascular and cerebrovascular death	Other deaths	t	p
n	53	27		
Age (years old)	62.57±11.82	60.26±10.68	0.85	0.40
Dialysis time (months)*	35.78±9.93	69.74±10.77	14.05	0.00
Creatinine (μmol/L)	643.82±20.89	653.09±25.46	1.74	0.09
Albumin (g/L)	33.64±7.81	31.40±7.49	1.23	0.22
HBG (g/L)*	83.01±12.74	92.75±13.60	3.16	0.00
TG (mmol/L)*	3.76±1.23	3.14±1.21	2.15	0.04
TC (mmol/L)*	8.57±1.52	7.24±1.06	4.05	0.00
LDL (mmol/L)	5.62±1.47	5.59±1.19	0.08	0.95
HDL (mmol/)*	1.13±0.29	1.45±0.36	4.22	0.00
P (mmol/L)	7.32±2.48	3.82±1.05	7.00	0.00

* p<0.05.

## DISCUSSION

Maintenance hemodialysis, is a well-established method of renal replacement therapy, which can significantly prolong the life of patients with chronic renal failure.^7^ However, patients with chronic renal failure on maintenance hemodialysis still suffer from a high mortality rate so far.^8^

Therefore, it is of great clinical value to investigate the causes and influencing factors of death in patients with chronic renal failure on maintenance hemodialysis, which can provide an objective reference for timely targeted interventions in clinical practice and reduce the mortality of patients. According to a study by Hiyamuta H et al.^9^, cardiovascular and cerebrovascular complications were the main risk factor for death in patients with chronic renal failure on maintenance hemodialysis. The main causes of death are heart failure, arrhythmia and sudden death.^10^

As shown in this study, among the 300 patients included, cardiovascular disease mortality was 40%, cerebrovascular disease mortality was 26%, and infection mortality was 23%. Logistic regression analysis showed that hypertension, high volume load and left ventricular hypertrophy were risk factors for the death of chronic renal failure patients on maintenance hemodialysis. It was shown in another study.^11^ that left ventricular hypertrophy had a close bearing on cardiovascular complications in patients on maintenance hemodialysis. Left ventricular hypertrophy is the most common Cardiac structural abnormality in patients on maintenance hemodialysis and is an independent risk factor for arrhythmia and ischemic heart disease in congestive heart failure.

Studies have shown that the mortality was significantly higher in maintenance hemodialysis patients with hyperphosphatemia than in patients with normal blood phosphorus, especially when serum P>2.49 mmol/L (CI: 1.04-2.07; P=0.03).^12^ In this study, Logistic regression analysis also showed that hyperphosphatemia was a risk factor for death in patients with chronic renal failure on maintenance hemodialysis (P=0.00), further confirming that controlling blood phosphorus level could effectively reduce the risk of death in patients on hemodialysis. It was demonstrated in a study by Hiyamuta et al.^13^ that serum phosphate level in hemodialysis patients is positively correlated with the risk of sudden death. In the presence of improved serum phosphorus levels and nutritional indicators, the risk of death in hemodialysis patients can be reduced.^14^

It was considered in the study^15^ that the levels of pro-atherogenic plaque formation factors such as TC and TG in hemodialysis patients were significantly increased, while HDL was decreased. Patients with MHD are at high risk of infection, and infection is one of the leading causes of death in MHD patients.^16^ Malnutrition is an important complication of MHD and affects the quality of life and the effect of dialysis in patients with MHD, and is also closely related to prognosis.^17^ Patients with MHD often develop hypoalbuminemia and anemia due to insufficient energy and protein intake, excessive dialysis or urinary protein loss, increased protein catabolism, and decreased erythropoietin.

It was confirmed in this study that the levels of HBG and HDL in patients with cardiovascular and cerebrovascular disease were significantly lower than those in the non-cardiovascular and cerebrovascular death group (P=0.00), while TG and TC were significantly higher than those in the non-cardiovascular and cerebrovascular death group (TG, P=0.02; TC, P=0.01). In multivariate analysis, male gender, age, DN, low hemoglobin, low albumin, and low blood calcium were associated with mortality in hemodialysis patients.^18^

### Limitations

Nevertheless, deficiencies can still be seen in this study: few cases are included, and there are still a considerable number of factors not included in the observation such as degree of obesity, body mass index, blood sugar level, patient’s lifestyle, exercise, etc. With the deepening of the study, we will further increase the sample content and observation indicators, and further enrich the research content, more detailed description of the causes of death and influencing factors of maintenance hemodialysis.

## CONCLUSION

The complications of maintenance hemodialysis in patients with chronic renal failure involve multiple organs and systems throughout the body. The main causes of death are cardiovascular and cerebrovascular diseases, infections, etc. To this end, the prevention and treatment of complications in patients with chronic renal failure on maintenance hemodialysis is of great importance. Adequate dialysis of patients, improvement of nutritional status and anemia of patients, and active control of blood pressure, hypervolemia, hyperlipidemia, and hyperphosphatemia are important measures to prevent cardiovascular and cerebrovascular diseases.

### Authors’ Contributions:

**XF and CB** designed this study, prepared this manuscript, are responsible and accountable for the accuracy and integrity of the work.

**JL** collected and analyzed clinical data.

**WL and FW:** Data analysis. significantly revised this manuscript.
